# Professional Secrecy

**Published:** 1919-06-14

**Authors:** 


### PROFESSIONAL SECRECY.

"The individual withers," says Tennyson, "and the race is more and more." If he lived in these days he would be justified in saying that the individual withers and the State, or at any rate the community, is more and more. In nearly everything the interest of the community, or what the numerical majority thinks is the interest of the community, and especially what it thinks is the interest of the members of the numerical majority, rides roughshod over the interest of individuals, who must "grin and abide." It is curious that in this general tendency to socialisation the primary duty of the individual to the community—the duty of defending it when attacked—is left optional. The individual must pay to the State roughly one-third of his earnings, and the more his earnings amount to the larger the proportion of them that he must pay to the State. He must submit to restrictions and privations in a hundred different matters, and the more valuable his services to the State the more of his reward is confiscated by the State, but the primary duty of defending the State from foreign foes is left to his own discretion to fulfil or neglect as he chooses.

The State regulates the pay the employer is to give to his workmen, and takes no account of the value of the services rendered in return, nor does the State concern itself that these services shall be honestly rendered. The State prescribes what education the parent is to give to his child and what he shall pay for the education of other people's children. The State provides medical attendance for its citizens, and soon, no doubt, it will prescribe the orthodox treatment for each disease; and when once the State begins to interfere actively with the normal conduct of its individual members, there is no knowing with what it will not intentionally interfere next; and, moreover, there is no knowing, and no heed on the part of legislators with what they have not already and unintentionally interfered.

There is an honourable tradition, more than two thousand years old, that transactions between the patient and his physician are confidential, and that the physician is bound by a strict rule of honour to reveal nothing that he may learn in his professional relations with his patients. This rule is now tacitly set aside by legislation. The physician is now bound by law to reveal certain items of knowledge that he may gain from his patient in professional consultations. He must reveal, if it is the fact that his patient is suffering from infectious or contagious disease. He must reveal, if it is a fact that his patient is mad.

How far are these revelations to extend, and, in particular, is the physician to report that his patient is suffering from venereal disease? And to whom may such revelation be made? It should seem that the question is to be determined by the balance of advantage and disadvantage. Venereal diseases, unlike most diseases that are now notifiable, do not expose to danger of infection those among whom the diseased person is merely present. Their communication requires actual contact, and usually close and prolonged contact, and the casual acquaintance, the fellow worker and fellow traveller, are in little danger. On behalf of the community at large, therefore, the notification of such diseases is scarcely justifiable. But the case is very different with respect to a prospective spouse. A healthy person who marries one suffering from venereal disease is practically certain to contract the disease, and the disease has dire consequences that endure for a lifetime. He or she who, knowingly suffering from venereal disease, contracts a marriage commits an infamous act that cannot be too strongly condemned, and that ought to be prevented even at great cost. The violation of professional secrecy is a very high price to pay for the prevention of such a wickedness—a wickedness that is now become a crime. It is a very high price, but it is not too high a price. If a physician, in the course of his professional attendance upon a patient, were to learn that a murder was contemplated, it would, no doubt, be his legal duty to violate the professional confidence, but whether it would be ethically right to do so is another matter, and one susceptible of argument; but that it is ethically right to reveal to a prospective spouse that the person with whom he or she contemplates marriage is suffering from venereal disease admits of no argument. To make the revelation would be in all probability to prevent a murder, a slow poisoning involving many years of suffering.

Thus we come round to the position in which experience places us all sooner or later, the position from which we see that circumstances alter cases, that no rule is universally binding, that conditions and qualifications may reverse the incidence and meaning of right and wrong; in short, that human affairs are infinitely complex, and are not to be regulated by rigid formulæ, even by formulæ prescribing what is honourable and what is not.

